# Patient and healthcare system factors associated with participation in municipal smoking cessation programs after hospitalization for atherosclerotic vascular disease in Norway: A prespecified analysis of two randomized controlled trials

**DOI:** 10.18332/tpc/236282

**Published:** 2026-10-13

**Authors:** Vilde Getz, Karin Pleym, Toril Dammen, Einar Husebye, Elise Sverre, Kari Peersen, Costas Papageorgiou, Harald Weedon-Fekjær, John Munkhaugen

**Affiliations:** 1 Department of MedicineDrammen HospitalDrammenNorway; 2 Department of Behavioral MedicineInstitute of Basic Medical Sciences, Faculty of Medicine, University of OsloOsloNorway; 3 Institute of Clinical Medicine, University of OsloOsloNorway; 4 Department of Research and InnovationDivision of Mental Health and Addiction, Oslo University HospitalOsloNorway; 5 Department of MedicineVestfold Hospital TrustTønsbergNorway; 6 Department of PsychologyUniversity of OsloOsloNorway; 7 Oslo Center for Biostatistics and Epidemiology, Research Support Services, Oslo University HospitalOsloNorway

**Keywords:** smoking cessation, atherosclerotic vascular disease, cardiac rehabilitation, municipal cessation program, patient and healthcare system factors

## Abstract

**Introduction:**

Persistent smoking after hospitalization for an atherosclerotic vascular event remains common, and only a small proportion attend cardiac rehabilitation or community-based lifestyle support, including smoking cessation programs. This study aimed to identify patient and healthcare system factors associated with participation in a municipal smoking cessation program among patients with atherosclerotic vascular disease included in a smoking cessation trial.

**Methods:**

A prespecified secondary analysis was conducted of two randomized cessation trials with patients recruited between 2021 and 2023 at three hospitals in Norway. Hospitalized patients with established atherosclerotic vascular disease who smoked at least one cigarette daily were randomized to motivational counseling with proactive referral to the municipal cessation program (intervention) or brief advice and contact information (control). Data were collected from medical records and self-report questionnaires. Associations between patient and healthcare system factors and program participation were explored using logistic regression analyses, adjusting for age, site and group allocation.

**Results:**

In all, 268 of 280 patients were eligible at 6 months (median age 65 years, 39% women). Participation in the cessation program was 42% (56/134) in the intervention and 9% (12/134) in the control group. Among participants, 57% (39/68) quit smoking, compared with 30% (59/200) among non-participants. In the total sample, older age (adjusted odds ratio, AOR=0.65 per 10 year increase; 95% CI: 0.47–0.90), living alone versus with others (AOR=0.30; 95% CI: 0.14–0.65), and lower versus higher education (AOR=0.40; 95% CI: 0.18–0.89) were negatively associated with participation. Analysis restricted to the intervention group yielded consistent findings.

**Conclusions:**

Participation in a municipal smoking cessation program among hospitalized patients with atherosclerotic vascular disease remained suboptimal, despite proactive motivational counseling and proactive referral. Older patients, those living alone, and patients with lower level of education were less likely to participate, highlighting the need for targeted referral strategies and tailored support for these groups.

## Introduction

Smoking remains prevalent among patients with atherosclerotic cardiovascular disease (ASCVD), with 50% of patients in European surveys and almost 60% in Norwegian cohorts continuing to smoke following hospitalization for an ASCVD event^1,2^.

Participation in multidisciplinary cardiac rehabilitation (CR) is associated with improved prognosis and a reduction in persistent smoking after an acute coronary event^3^ and is therefore strongly recommended. However, in routine clinical practice, only a small proportion of eligible patients enroll^3,4^. In addition, smokers are less likely to attend and complete CR than non-smokers^5^. Consequently, alternative and complementary programs have been called for, preferably anchored in existing national and local health services^6^.

Barriers to CR participation include system factors, such as long travel distances, transportation issues, lack of referral, and limited availability, as well as patient factors such as lower socioeconomic status, older age, low awareness, and comorbidities^7^. Psychosocial factors, including mental distress and social isolation, further impede lifestyle changes and program engagement^8^. Community-based services, such as Norway’s Healthy Life Centers, provide smoking-cessation support. Although proactive referral to smoking cessation programs appears to be effective^9^, participation rates vary, and attendance at such services among cardiovascular populations remains suboptimal^10^. This underscores the need for a better understanding of which patients access and use these services. Qualitative work suggests that many smokers have limited knowledge of services, fear stigma, or face practical barriers such as work and time pressures, referral delays, and transport difficulties^11^. Predictors of engagement in behavioral smoking cessation programs include readiness to quit, prior quit attempts, age, cohabitation, education level, smoking intensity, and insomnia, while anxiety, depression, and low treatment engagement predict non-participation^12-18^. However, most evidence is from general smoking cohorts, with limited evidence on engagement in municipal cessation programs among patients with established cardiovascular disease.

In two recent hospital-based smoking cessation trials among patients with ASCVD, motivational counseling combined with proactive referral significantly increased participation in the municipal Healthy Life Center^19^ and doubled long-term quit rates^20^. However, half of eligible patients did not attend the cessation program^19^. Building on this, we here explore prespecified patient-level and healthcare system factors associated with participation in municipal smoking cessation programs among patients with ASCVD enrolled in a randomized cessation trial after hospitalization for a vascular event.

## Methods

### Design and population

This was a prespecified secondary analysis of two prospective, randomized, open-label, blinded endpoint evaluation (PROBE) intervention trials involving a total of 278 participants^19,20^. A proof-of-concept study (n=58) evaluated the effect of a hospital-based smoking cessation intervention on participation rates at municipal cessation programs and the use of smoking cessation medication after 6 months^19^. Later, a main study (n=220) assessed the impact of the intervention on smoking cessation after 6 and 12 months^20^.

Participants were recruited from three hospitals within the Vestre Viken Trust, a large hospital trust in South-Eastern Norway. The three hospitals serve approximately 7% of Norway’s population and include both urban and rural municipalities, yielding a geographically and sociodemographically diverse regional sample. Patients with confirmed ASCVD, admitted for either acute or planned vascular events, including coronary artery disease (CAD), cerebrovascular disease, peripheral artery disease, valvular disease, arrhythmias, or heart failure, who smoked at least one cigarette daily before admission, were consecutively invited to participate between January 2021 and October 2023. Notably, patients were included regardless of their motivation to quit smoking. Eligibility criteria included being over 18 years old, residing or working in the Vestre Viken region, and being able to sign informed consent. Exclusion criteria included a life expectancy below 12 months, stage 4 chronic kidney disease, or conditions making participation unethical (e.g. psychosis, alcohol abuse, or dementia) (Figure 1). Randomization occurred after written informed consent was obtained.

**Figure 1:**
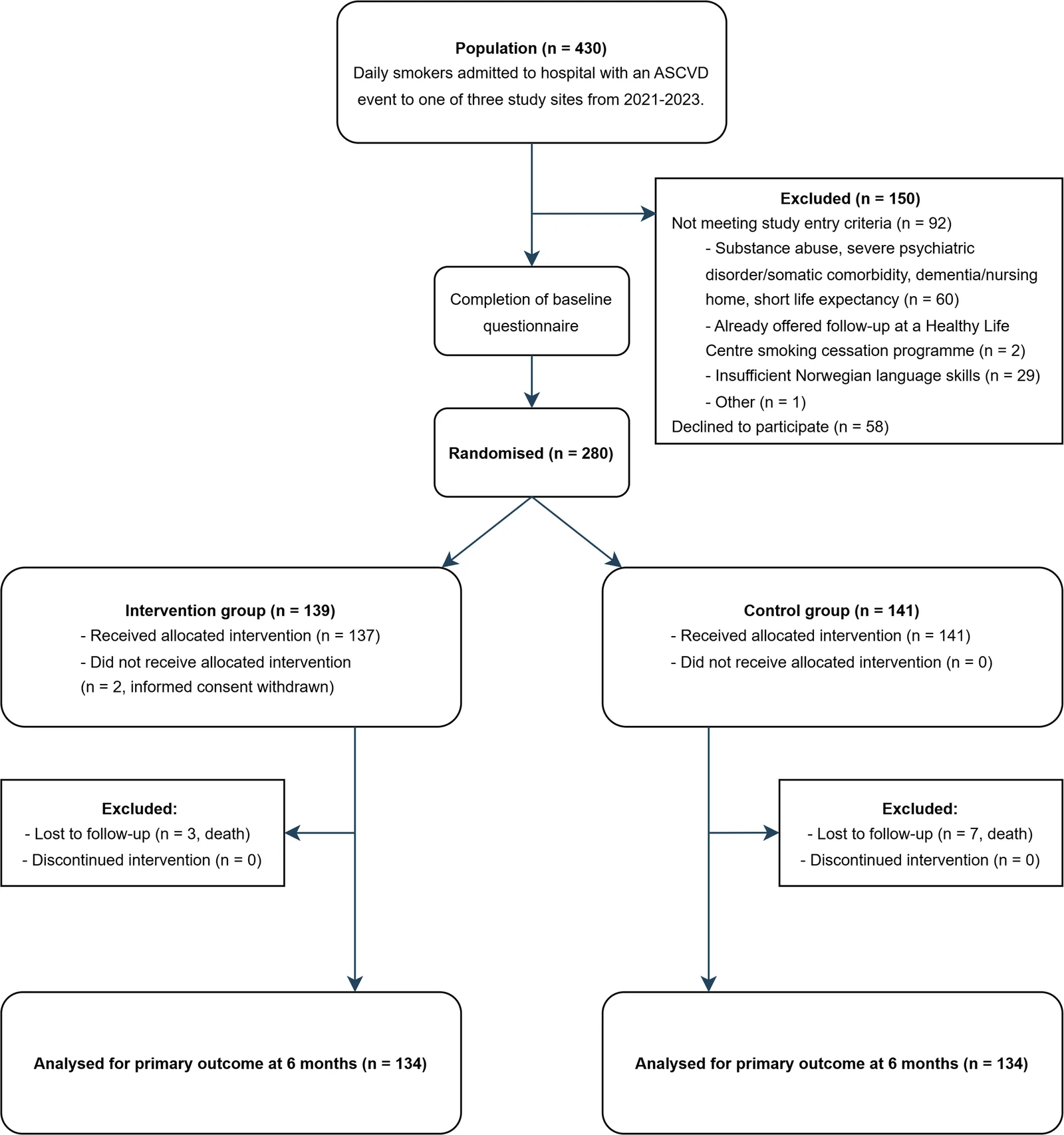
Flowchart of participant progression in two randomized smoking cessation trials among patients hospitalized for a vascular disease event, Norway, 2021–2023. ASCVD: atherosclerotic cardiovascular disease.

The proof-of-concept study (Identifier: NCT04772144) and the main study (Identifier: NCT05049174), as well as the present analysis, were preregistered on ClinicalTrials.gov and followed the CONSORT guidelines. The trials were evaluated by the Regional Ethics Committee (REC identifiers 202686 and 270267) and the local Data Protection Officer (number 21/00348–1 / and 21/07103-1/005). More in-depth details of the methods and primary results of the two randomized intervention trials have been reported in their respective articles^19,20^.

### The municipal smoking cessation program

Smoking cessation programs were delivered by national low-threshold Healthy Life Centers in most municipalities within the Vestre Viken Trust catchment area. Municipal health services are accessible to all and support lifestyle changes^21^. They offer various follow-up options based on participants’ preferences, including in-person, digital, or telephone meetings, as well as individual counseling or group sessions. The cessation program was developed by the Directorate of Health in Norway, based on recommendations for combined behavioral follow-up and the use of smoking cessation medication^22^. These recommendations allow adaptation to local requirements and resources. Consequently, all employees are trained in motivational interviewing and in the proper use of cessation drugs. The Directorate of Health initiated a pilot project that funded free cessation medication, primarily nicotine replacement therapy, for all participants in the smoking cessation program from September 2020 to December 2023^23^. Thus, all study participants had the option to receive vouchers for free cessation medication for up to 3 months, which could be collected at the pharmacy. The cessation program included follow-up for preparation, cessation, and maintenance, with weekly sessions lasting five to 7 weeks. If desired, further follow-up could be offered after the official program ended^22^.

### Intervention

In both trials, participants were randomly assigned in a 1 : 1 ratio to the intervention or control group, using a randomization sequence executed by an independent statistician. Non-transparent envelopes, prepared outside the research team, were used for allocation. Study personnel involved in recruitment and outcome assessment were blinded to group assignments.

The intervention group received a 30 min in-hospital conversation with a study nurse using motivational interviewing techniques. Upon discharge, patients were referred directly by telephone to the municipal smoking cessation program, making it an opt-out intervention. Participants were then contacted within 2 weeks to promote their participation in the municipal cessation program.

The control group received the current clinical practice at the relevant hospital along with a brochure containing information on smoking cessation and contact information for the municipal smoking cessation program.

For both groups, participation in the municipal program was optional, and vouchers for free cessation medications were provided only to those who attended. In the main study, a letter was also sent to the participants' general practitioner, informing them of their inclusion in the study and the cessation program.

### Outcome assessment

The main outcome in this prespecified secondary study was participation in the municipal smoking cessation program (yes, no), defined as participation in at least one individual or group session (in-person, digital or telephone) within 6 months after randomization, as recorded in the municipal Healthy Life Center medical records.

### Covariates

Prespecified data from medical records were retrospectively collected. From the medical records at the municipal centers, the numbers, types (individual vs group sessions), and delivery mode (telephone, digital, or physical) of the consultations were recorded. We calculated the travel distance in kilometers along roads from the participant’s home to the local center serving the patients’ area.

From hospital medical records, we recorded age, sex, and index diagnosis, defined as the vascular event that caused hospitalization. Coronary heart disease was defined as angina or myocardial infarction, and cerebrovascular disease as stroke or transient ischemic attack. Somatic comorbidity was assessed using the Charlson Comorbidity Index, a scoring system that quantifies a patient’s comorbidity to estimate 10 year survival, with higher scores reflecting greater morbidity and mortality risk. Psychiatric comorbidities were identified through records of anxiety and depression diagnoses prior to the index event. We also used information on study inclusion in June or July, when summer closures or reduced staffing may delay initial contact from the smoking cessation program.

Clinical and psychosocial factors were collected through validated self-reported questionnaires administered during hospitalization for the index event and completed before randomization. Socioeconomic factors included education level and living arrangements. Low level of education was defined as completion of primary or secondary school only. Psychosocial variables included symptoms of depression and anxiety, assessed with the Hospital Anxiety and Depression Scale (HADS), with a score of ≥8 points shown to provide the best sensitivity and specificity; sleep duration, measured as the mean hours and minutes of sleep per night over the past week; insomnia (yes, no), as evaluated with the Bergen Insomnia Scale; and Type D Personality, assessed with the DS-14 questionnaire. Motivation to quit was assessed using a Likert scale ranging from 0 (no consideration of quitting) to 10 (readiness to take action to quit), in accordance with the contemplation ladder. Readiness for smoking cessation was assessed using a modified stage of change algorithm, categorizing individuals into precontemplation (no intent to quit within 6 months), contemplation (thinking about quitting in the next 6 months), or preparation (intends to quit in the next 30 days with a past quit attempt of at least 24 h). Nicotine dependence was evaluated using the Fagerström test for nicotine dependence, a six-item questionnaire with scores ranging from 0 to 10, with higher scores representing higher dependence.

Structured telephone interviews were conducted at 1, 3, and 6 months post-inclusion to gather data on CR participation (yes, no), use of cessation drugs, and smoking status. A current smoker was defined as having smoked >5 cigarettes since beginning the cigarette abstinence period. In the main study, self-reported abstinence was verified by measuring exhaled carbon monoxide (CO).

### Statistical analysis

Data are presented as medians with interquartile range (IQR) for continuous variables and frequencies with percentages for categorical variables. Analyses included all randomized patients eligible for analysis within 6 months of follow-up. Associations between participation in the municipal cessation program and relevant covariates were explored using logistic regression. Crude and multivariable-adjusted odds ratios (AORs) with 95% confidence intervals (CIs) were calculated. The significance level was set at p<0.05. Separate analyses were performed for the intervention group. A pairwise correlation matrix was constructed to identify potential strong correlations that might influence the interpretation of the results. Missing data in covariates were handled using a complete-case analysis in the primary models. As a sensitivity analysis, we repeated the regression analyses with imputed data using multiple imputation by chained equations (MICE). All analyses were conducted in STATA version 18 (StataCorp LLC, College Station, TX, USA).

## Results

### Characteristics at baseline

Of 430 patients hospitalized with ASCVD and screened for participation, 92 (21%) met an exclusion criterion, and 58 (14%) declined participation. Of the 280 randomized participants, two withdrew their consent, and ten died within 6 months of follow-up, leaving 268 participants available for analysis (Figure 1). The median age was 65 years (IQR: 58–72), and 39% were women. Coronary heart disease (49%), peripheral vascular disease (17%), and cerebrovascular disease (15%) were the most prevalent index diagnoses. The majority had a low level of education (78%), and 31% were employed. Most participants had smoked for over 20 years (90%) but reported high motivation to quit, with a median score of 8 out of 10 (Table 1). Among psychological factors, 35% reported having clinically significant anxiety and/or depressive symptoms, and 45% reported symptoms of insomnia (Table 1). Characteristics of the group that answered <80% of the questionnaires are presented in Supplementary file Table 1.

**Table 1:** Baseline characteristics according to participation in the municipal smoking cessation program in two randomized smoking cessation trials among patients hospitalized for a vascular disease event, Norway, 2021–2023 (N=268)

Characteristics	Participants (N=68)	Non-participants (N=200)	Total (N=268)
**Age at index event** (years), median (IQR)	61 (56–67.5)	67 (60–73)	65 (58–72)
**Female**	31 (45.6)	73 (36.5)	104 (38.8)
**Low level of education** ^**a**^	40/59 (67.8)	137/168 (81.6)	177/227 (78.0)
**Employed**	20/59 (33.9)	51/171 (29.8)	71/230 (30.9)
**Living alone**	15/59 (25.4)	70/171 (40.9)	85/230 (37.0)
**Index cardiovascular event**			
Coronary heart disease^b^	40 (58.8)	91 (45.5)	131 (48.9)
Cerebrovascular disease^c^	13 (19.1)	26 (13.0)	39 (14.6)
Peripheral vascular disease	8 (11.8)	38 (19.0)	46 (17.2)
Other cardiovascular diseases	7 (10.3)	45 (22.5)	52 (19.4)
**Smoking status**			
Duration of smoking >20 years	56/59 (94.9)	150/170 (88.2)	206/229 (90.0)
Cigarettes per day >20	12/59 (20.3)	26/168 (15.5)	38/227 (16.7)
Living with a smoking partner	23/59 (39.0)	37/170 (21.8)	60/229 (26.2)
**Nicotine dependence^g^**, median score (IQR)	4 (3–5)	4 (2–5)	4 (2–5)
**Readiness for smoking cessation^f^**			
Preparation phase	25/57 (43.9)	61/166 (36.8)	86/223 (38.6)
Precontemplation phase	16/57 (28.1)	56/166 (33.7)	72/223 (32.3)
**Motivation to quit^e^**, median (IQR)	8 (6–10)	8 (6–10)	8 (6–10)
**Health status**			
Insomnia^i^	25/50 (50.0)	53/122 (43.4)	78/172 (45.4)
Average duration of sleep in hours, median (IQR)	7 (6–8)	7 (6–7.8)	7 (6–8)
HADS anxiety and/or depression score ≥8	16/50 (32)	50/140 (35.7)	66/190 (34.7)
Anxiety and/or affective disorder (hospital record diagnosis)	17 (25.0)	32 (16.0)	49 **(18.3)**
Type D personality^h^	9/48 (18.8)	21/119 (17.7)	30/167 (18.0)
**Charlson comorbidity sum score^d^**, median (IQR)	3 (2–4)	4 (3–6)	4 (3–5.5)
**Healthcare system factors**			
**Travel distance^j^** (km), median (IQR)	5.5 (2.7–12.1)	8.2 (3.8–13.7)	8 (3.5–13.3)
**Inclusion in June or July^k^**	13 (19.1)	24 (12.0)	37 (13.8)
**Participation in CR**	17/60 (28.3)	27/154 (17.5)	44/214 (20.6)

Data are given as n (%); for variables with missing values, the fraction of respondents is presented. Participants who died within the first 6 months after inclusion were excluded. HADS: Hospital Anxiety and Depression Scale. CR: cardiac rehabilitation. IQR: 25th-75th percentile.

a Low level of education was defined by completion of primary or secondary school only.

b Coronary heart disease is defined as angina or myocardial infarction.

c Cerebrovascular disease is defined as stroke or transient ischemic attack.

d Charlson comorbidity sum score, a scoring system to quantify a patient’s somatic comorbidity and to predict a 10-year survival rate, with higher scores reflecting higher morbidity and mortality risk.

e Motivation to quit was assessed using a Likert scale ranging from 0 (low motivation) to 10 (high motivation).

f Readiness to quit was measured using a modified stage of change algorithm, categorizing individuals into precontemplation (no intent to quit within 6 months), contemplation (thinking about quitting in the next 6 months), or preparation (intends to quit in the next 30 days with a past quit attempt of at least 24 h).

g Nicotine dependence was assessed by Fagerström test, a six-item questionnaire with scores ranging from 0 to 10: very low = 0–2, low = 3–4, moderate = 5, high = 6–7 and very high = 8–10.

h Type D personality was measured by the DS-14 questionnaire.

i Insomnia was measured by Bergen Insomnia Scale.

j Travel distance along the road network to the nearest municipal smoking cessation program serving the patients’ area.

k Study inclusion in June or July, when summer closure or reduced staffing may delay initial contact from the smoking cessation program.

### Participation in the municipal cessation program and smoking cessation

In all, 68 of 268 (25%) patients participated in the municipal smoking cessation program, with 56/134 (42%) from the intervention group and 12/134 (9%) from the control group. Of these, 57% (39/68) had quit smoking at 6 months. Among the 29 who continued smoking, there were fewer follow-up consultations and more group sessions compared to individual sessions than among those who quit (Table 2). Among those not attending the cessation program, 30% (59/200) quit smoking. Compared with both non-participants who continued to smoke and program participants, these unassisted quitters were characterized by lower nicotine dependence (median Fagerström score 3 vs 4 and 5), higher motivation to quit (median 10 vs 7 and 8), a higher proportion with coronary heart disease as the index diagnosis (61% vs 39 and 59%), and a lower proportion with insomnia (37% vs 46 and 50%), and anxiety and/or depression (HADS anxiety/depression score ≥8 : 28% vs 39 and 32%; hospital record diagnosis of anxiety/depression: 7% vs 20 and 25%) (Supplementary file Table 2).

**Table 2:** Characteristics of the municipal smoking cessation program by 6-month smoking status in two randomized smoking cessation trials among patients hospitalized for a vascular disease event, Norway, 2021–2023

Characteristics	Quit smoking (N=39) n (%)	Persistent smoking (N=29) n (%)	Total (N=68) n (%)
**Frequency of participation**, median (IQR)	5 (3–6)	4 (2–6)	4 (3–6)
1–4	19 (49)	17 (59)	36 (53)
≥5	20 (51)	12 (41)	32 (47)
**Digital vs physical participation**			
Digital/telephone	13 (33)	4 (14)	17 (25)
Physical	13 (33)	17 (59)	30 (44)
Combination	13 (33)	8 (28)	21 (31)
**Individual vs group counseling**			
Individual	30 (77)	10 (35)	49 (72)
Group	9 (23)	19 (65)	19 (28)
**Number of received vouchers^a^**	32 (82)	26 (90)	58 (85)
1	5 (13)	9 (31)	14 (21)
>1	27 (69)	17 (59)	44 (65)

a One voucher covered cessation medication for a three-month duration. IQR: 25th-75th percentile.

### Patient and healthcare system factors associated with program participation

In analyses adjusted for age, site, and group allocation, factors statistically significantly and negatively associated with participation were older age (AOR=0.65 per 10 year increase; 95% CI: 0.47–0.90); living alone (AOR=0.30; 95% CI: 0.14–0.65; reference: living with others); and low education (AOR=0.40; 95% CI: 0.18–0.89; reference: high education). There was a tendency towards non-participation among patients with a higher somatic comorbidity score; but the association was not statistically significant in this moderate-sized study (AOR=0.82 per point on the Charlson score; 95% CI: 0.65–1.04) (Table 3). None of the possible associations between psychological or sleep-related factors and program participation reached statistical significance in our study (Table 3). In adjusted analyses: insomnia (AOR=1.25; 95% CI 0.58–2.68; reference: no insomnia); shorter sleep duration per hour (AOR=0.93; 95% CI: 0.71–1.23); type D personality (AOR=0.98; 95% CI: 0.36–2.62; reference: no type D personality); self-reported anxiety and/or depression (HADS score ≥8; AOR=0.80; 95% CI: 0.36–1.77; reference: HADS score <8); and hospital-recorded anxiety and/or affective disorder (AOR=1.60; 95% CI: 0.73–3.51) all showed non-significant associations with program participation (Table 3). In adjusted analyses excluding patients who quit smoking without participating in the smoking cessation program, all statistically significant factors showed stronger associations with participation, and a higher somatic comorbidity score was statistically significantly and negatively associated with participation (Supplementary file Table 3).

**Table 3:** Odds ratios for participation in the municipal smoking cessation program in the total population from two randomized smoking cessation trials, six months after hospitalization for a vascular disease event, Norway, 2021–2023 (N=268)

Factors	OR (95% CI)	p*	AOR (95% CI)	p*
**Patient**				
Age per 10 years	0.61 (0.45–0.81)	0.001	0.65 (0.47–0.90)^a^	0.010^a^
Female gender	1.46 (0.84–2.55)	0.185	1.16 (0.62–2.19)	0.639
Living alone	0.49 (0.25–0.95)	0.035	0.30 (0.14–0.65)	0.002
Low level of education^b^	0.48 (0.24–0.93)	0.030	0.40 (0.18–0.89)	0.025
Employed	1.21 (0.64–2.27)	0.559	0.52 (0.23–1.19)	0.119
Charlson comorbidity score^c^ per 1.0 point	0.74 (0.63–0.87)	<0.001	0.82 (0.65–1.04)	0.101
MI or stroke as index diagnosis	1.30 (0.75–2.26)	0.353	1.11 (0.59–2.09)	0.751
Motivation to quit smoking per 1.0 point^d^	1.07 (0.95–1.22)	0.256	1.00 (0.87–1.16)	0.972
Moderate to very high nicotine dependence^e^	0.81 (0.41–1.57)	0.526	0.74 (0.34–1.60)	0.444
Insomnia^f^	1.30 (0.67–2.52)	0.433	1.25 (0.58–2.68)	0.570
Shorter sleep duration (hours)	0.99 (0.78–1.25)	0.904	0.93 (0.71–1.23)	0.622
Type D personality^g^	1.08 (0.45–2.56)	0.867	0.98 (0.36–2.62)	0.960
HADS anxiety and/or depression score ≥8	0.85 (0.43–1.68)	0.636	0.80 (0.36–1.77)	0.584
Anxiety and/or affective disorder (hospital record diagnosis)	1.75 (0.90–3.41)	0.100	1.60 (0.73–3.51)	0.243
**Healthcare system**				
Travel distance^h^ per 10 km	0.84 (0.63–1.14)	0.269	0.85 (0.61–1.18)	0.325
Inclusion in June/July^i^	1.73 (0.83–3.63)	0.145	1.81 (0.77–4.24)	0.171
Participation in CR	1.86 (0.93–3.74)	0.082	1.37 (0.59–3.18)	0.462

* P-values, estimated from logistic regression with participation in the municipal smoking cessation program within 6 months after hospitalization for a vascular disease event (yes, no) as the dependent variable. Reference categories: female vs male, living alone vs with others, low vs high level of education, employed vs unemployed, MI/stroke vs other vascular index diagnoses, moderate to very high vs low nicotine dependence, insomnia (yes, no), type D personality (yes, no), HADS anxiety and/or depression score ≥8 vs <8, anxiety and/or affective disorder (yes, no), inclusion in June/July vs other months, participation in CR (yes, no).

a Adjusted for site and group allocation. MI: myocardial infarction. HADS: Hospital Anxiety and Depression Scale. CR: cardiac rehabilitation.

b Low level of education was defined by completion of primary or secondary school only.

c Charlson comorbidity sum score, a scoring system to quantify a patient’s somatic comorbidity and to predict a 10-year survival rate, with higher scores reflecting higher morbidity and mortality risk.

d Motivation to quit was assessed using a Likert scale ranging from 0 (low motivation) to 10 (high motivation).

e Nicotine dependence was assessed by the Fagerström test, a six-item questionnaire with scores ranging from 0 to 10: very low = 0–2, low = 3–4, moderate = 5, high = 6–7 and very high = 8–10.

f Insomnia was measured by Bergen Insomnia Scale.

g Type D personality was measured by the DS-14 questionnaire.

h Travel distance along the road network to the nearest municipal smoking cessation program serving the patients’ area.

i Study inclusion in June or July, when summer closure or reduced staffing may delay initial contact from the smoking cessation program.

AOR: adjusted odds ratio; adjusted for age, site and group allocation.

The median travel distance to the municipal cessation center was 8 km (IQR: 3.5–13.3), and only 14% (n=37) of participants were included during the summer holiday period. None of these healthcare system factors was statistically significantly associated with program participation, but the uncertainty was large (Table 3).

Analysis of patient and healthcare system factors restricted to the intervention arm (n=134) showed comparable results (Supplementary file Table 4). Pairwise correlations between patient and healthcare system factors are presented in Supplementary file Table 5.

### Sensitivity analyses

The analyses in the imputed dataset did not differ significantly from the primary results (Supplementary file Table 6).

## Discussion

In this prespecified analysis of two randomized trials involving patients hospitalized with vascular disease, participation in the intervention group remained modest at 42%, despite in-hospital motivational counseling and a proactive referral strategy. Nevertheless, this participation rate was substantially higher than the 9% in the control group. In the total sample, participation was more strongly associated with age, comorbidity, and social context than with psychological distress or healthcare system factors. Specifically, individuals living alone, of older age, and with a lower level of education were associated with lower participation. As expected, all statistically significant factors showed stronger associations with participation when patients who quit unassisted were excluded. Their characteristics suggest that patients who successfully quit smoking without assistance tend to have fewer clinical and psychosocial barriers and higher motivation to quit, whereas smoking cessation programs increasingly serve a more vulnerable subgroup in whom social and clinical barriers are more pronounced.

### Participation in municipal smoking cessation programs

Norwegian municipal Healthy Life Centers are designed as low-threshold services for lifestyle change, yet few eligible patients are informed about or attend these programs in routine care. In the catchment area of this study, previous findings reveal that only 2.4% of cessation program participants reported the hospital as their source of information^23^. In line with previous studies, the proactive referral strategy increased participation to 42%, as objectively verified by medical records, compared with usual care or self-referral^9^. Nonetheless, most smokers did not attend the municipal cessation program, and 30% of these quit unassisted, consistent with European data showing frequent unsupported quit attempts^24^.

Hospitalization for an acute vascular event may be effective in promoting smoking cessation^25^. In a nurse-coordinated cessation trial among CAD patients, most of the successful quitters quit immediately after their hospital stay^10^. However, for the substantial proportion of patients who do not succeed in quitting on their own, proactive referral appears necessary but not sufficient.

### Patient and healthcare system factors

Older patients were less likely to participate than younger patients. While some studies link younger age to participation, these often involve populations with lower mean ages^13,17,18^. Other studies have found lower participation rates in CR and smoking cessation programs after the age of 60–65 years^4,7,14^. Comorbidities have also been identified as a barrier to CR participation^7,26^. In our study, higher comorbidity was negatively associated with program participation and also strongly correlated with older age.

Several mechanisms may explain low participation among older patients with comorbidities. Older smokers might be a more selective persistent smoking group, as they have not yet quit^27^. Qualitative studies describe older smokers who feel too old to quit or believe it is too late to change their behaviour^11^. Perceived poor health may also reduce motivation to quit smoking^28^.

For older adults, the most effective cessation strategy is likely multimodal, combining pharmacotherapy with behavioral counseling, especially when delivered face-to-face^29^. Supporting this, we found higher cessation rates among those receiving individual follow-up than among those participating in group sessions. In CR, home-based multimodal programs have been suggested for older and comorbid patients rather than center-based services^30^. These programs have also shown promising effects on smoking cessation and should be considered for older ASCVD patients who continue smoking^31^.

Patients living alone were less likely to participate than those living with others. Although living alone is not synonymous with social isolation or lack of social support^32^, living status has been associated with both CR attendance and participation in smoking cessation programs^13,26,32^. Having no partner has been associated with non-participation and drop-out from CR^26^, whereas social support from family members, particularly when the support person engages in the behavior change themselves, has been associated with CR engagement^32^. Patients living alone may lack the day-to-day support for behavioral change that a spouse or partner can provide, including both mental and practical help such as transport or digital assistance.

A recent Cochrane review found no clear effect of social network or support interventions on cardiovascular mortality or smoking cessation^33^. The studies evaluated multicomponent CR or secondary prevention interventions in which smoking was one of several targeted behaviors, and where social support, typically from family or healthcare professionals, was a central component^33^. In contrast, municipal Healthy Life Centers offer group-based programs that do not explicitly aim to enhance social support but instead emphasize structured sessions for smoking cessation. Qualitative studies indicate that participants in these lifestyle programs value the group as a source of social support and a sense of belonging, which, in turn, increases motivation for behavioral change^34^. Our study showed that those in individual sessions were more likely to quit. However, we did not collect information on which counseling modes (group-based, individual, or both) were available at each center, leaving the results uncertain. Nevertheless, this suggests that the key challenge may not be changing the existing program structure but rather ensuring access to group counseling and developing better strategies to engage patients who live alone, emphasizing the opportunity to join a group with shared goals while also offering flexible, individually tailored support options.

In the total study population, low level of education was negatively associated with participation in the cessation program. Low level of education is a proxy for low socioeconomic status (SES) and a major barrier to smoking cessation and CR participation^7,26,35^. These patients may have fewer resources and skills to engage in health-promoting behaviors, contributing to a higher burden of ASCVD risk factors^26^. It has therefore been recommended that this patient group be prioritized for tailored counseling on their cardiac risk profile and the benefits of lifestyle modification, as they have an elevated risk of both developing cardiovascular disease and of non-adherence to healthcare interventions^7,26^.

None of the psychological factors, including sleep parameters, were significantly associated with program participation. Regarding anxiety and depression, self-reported significant symptoms tended to decrease the probability of participation, whereas a diagnosis of at least one anxiety or affective disorder registered in hospital records, increased it. One explanation may be that patients with a recorded diagnosis have previously sought and received treatment and therefore have fewer current symptoms, more positive experiences with healthcare, and a higher general willingness to seek help for other issues, including smoking cessation. This interpretation is partly in line with previous literature showing that depressive symptoms can act as a barrier to participation and increase dropout from CR, while also reflecting that findings on anxiety and depression as predictors of participation are not entirely consistent^26,36^. Finally, it might be that self-reported symptoms and recorded diagnoses did not capture the underlying psychological phenomena most relevant to participation decisions.

None of the healthcare system factors, including inclusion during the summer holiday period, travel distance to the cessation program, or participation in CR, was statistically significantly associated with program participation. Although the point estimates suggested that these factors might influence participation, wide confidence intervals prevented firm conclusions. Our findings do not provide clear evidence that travel distance or inclusion during the summer holiday period substantially limit access to the municipal cessation programs offering digital, telephone, and in-person counseling. Unmeasured organizational features, such as scheduling flexibility, language adoption, and staff capacity, may also influence participation, particularly among vulnerable subgroups. Moreover, these findings come from a setting with universal health coverage and well-developed municipal services. In health systems with fewer structural resources, social and clinical barriers similar to those identified here are likely to exist alongside financial and access barriers, implying that our estimates may underestimate the difficulties of achieving equitable participation in less supportive healthcare environments.

### Implications

Patients with atherosclerotic vascular disease, who were older, single, of low level of education, were less likely to participate in municipal cessation services despite in-hospital motivational cessation counseling with proactive referral. To address these disparities, healthcare systems may need to consider prioritizing these high-risk groups, for example through repeated offers of counseling and invitations to municipal programs, potentially in collaboration with general practitioners. These potential strategies warrant further assessment. In addition, the counseling may be more tailored and intensified to address perceived age-related barriers and vulnerable populations without caregiver support and with low socioeconomic status.

### Limitations

Several limitations need to be acknowledged. This study was conducted within a Norwegian healthcare system characterized by universal coverage, access to CR, and well-developed municipal health services, which may limit generalizability to other settings. Patients who were unable to understand Norwegian, had severe psychiatric comorbidity, or had short life expectancy were excluded, which limits transferability to individuals facing linguistic and cultural barriers, and to those who are most mentally and medically fragile.

The study used an open-label design, although endpoint assessment was blinded. For participation in the municipal smoking cessation program, we lacked data on how many patients the municipal program was unable to reach and on the number of contact attempts made. Municipal Healthy Life Centers have the flexibility to adapt cessation programs to their capacity and local needs, which may have resulted in variation in the offers provided across municipalities.

There may be inaccuracies in the program participation data obtained from the municipal health services. We also did not gather systematic data on patients’ perceptions or reasons for non-participation. Qualitative work suggests that beliefs about treatment, previous experiences, and perceived support are important for cessation decisions^11^; combining such data with quantitative predictors would provide a more comprehensive understanding.

Overall, we had limited statistical power in subgroup analyses, which, for some factors, reduces the certainty of our findings and may have led to a type II error. There were missing data, especially for the psychological variables. Respondents who completed <80% of the questionnaire were more likely to be women and had a higher median age than those who answered ≥80%. However, the analysis of the imputed dataset did not differ significantly from the primary analysis. There was potential collinearity between sociodemographic and social-context variables. Nevertheless, all correlations were in the expected direction, with several variables likely partly measuring the same clinical effect. Psychiatric comorbidity and psychosocial factors derived from routine records may be underreported, and hospital record diagnoses cannot distinguish between current and previous episodes.

## Conclusions

Participation in municipal smoking cessation programs among patients hospitalized for atherosclerotic vascular disease was significantly higher in the intervention group receiving motivational interviewing and proactive referral than in the control group, but remained suboptimal, with fewer than half attending. Non-participation was associated with older age, comorbidities, and social context rather than with psychological distress and healthcare system factors. In contrast, patients who quit smoking without program support appeared to face fewer clinical and psychosocial barriers and to be more motivated. These findings may inform healthcare planning by helping to identify high-risk subgroups unlikely to access existing services and who may require more targeted referral strategies, while suggesting that less resource-intensive approaches could be sufficient for patients who are able to quit unassisted.
