# Supplementary material for: Patient and healthcare system factors associated with participation in municipal smoking cessation programs after hospitalization for atherosclerotic vascular disease in Norway: A prespecified analysis of two randomized controlled trials
Source: Tob Prev Cessat. 2026 Oct 13;12:10.18332/tpc/236282. doi: 10.18332/tpc/236282 (PMC13662561; doi:10.18332/tpc/236282)
Supplement: Supplementary file 1 [file TPC-12-61-s001.pdf]

TOBACCO PREVENTION AND CESSATION

Supplementary file

© 2026 Getz V. et al.

DOI:
10.18332/tpc/236282

The content has been provided by the author(s) and has not been reviewed, verified, or endorsed by European Publishing. It may not have undergone peer review. The views, opinions, and recommendations expressed are solely those of the author(s) and do not necessarily reflect the position of European Publishing. European Publishing accepts no responsibility or liability for any consequences arising from the use of, or reliance on, this content.

Supp Table 1. Clinical characteristics among those with and without missing data in the total population from two randomized smoking cessation trials among patients hospitalized for a vascular disease event, Norway, 2021-2023 (n = 268)

	**Missing > 20 % (n = 68)**	**Answered > 80 % (n = 200)**
Allocated to the intervention group, n (%)	37 (54.4)	97 (48.5)
Age (years), median (Q1-Q3)	69 (64-75)	64 (57-71)
Female gender, n (%)	34 (50)	70 (35)
Charlson comorbidity score[a], median (Q1-Q3)	4 (3-6)	4 (3-5)
MI or stroke as index diagnosis, n (%)	24 (35.3)	97 (48.5)
Anxiety diagnosis, n (%)	6 (8.8)	26 (13.0)
Affective disorder diagnosis, n (%)	8 (11.8)	35 (17.5)
Inclusion in June/July[b], n (%)	13 (19.1)	24 (12.0)
Participation in the municipal cessation program, n (%)	15 (22.1)	53 (26.5)

MI: Myocardial Infarction.

[a]Charlson comorbidity sum score, a scoring system to quantify a patient's somatic comorbidity and to predict a 10-year survival rate, with higher scores reflecting higher morbidity and mortality risk.

[b]Study inclusion in June or July, when summer closure or reduced staffing may delay initial contact from the smoking cessation program.

Supp table 2. Baseline characteristics according to participation in the municipal smoking cessation program and smoking status in two randomized smoking cessation trials, six months after hospitalization for a vascular disease event, Norway, 2021-2023 (n = 268)

	**Non-participants, quit smoking (n = 59)**	**Non-participants, continued smoking (n = 141)**	**Participants (n = 68)**
Age at index event (years), median (Q1-Q3)	67 (60-73)	67 (60-74)	61 (56-67.5)
Female, n (%)	24 (40.7)	49 (34.8)	31 (45.6)
Low education[a], n (%)	38/47 (80.9)	99/121 (81.8)	40/59 (67.8)
Employed, n (%)	20/49 (40.8)	31/122 (25.4)	20/59 (33.9)
Living alone, n (%)	14/49 (28.6)	56/122 (45.9)	15/59 (25.4)
Index cardiovascular event			
Coronary heart disease[b], n (%)	36 (61.0)	55 (39.0)	40 (58.8)
Cerebrovascular disease[c], n (%)	7 (11.9)	19 (13.5)	13 (19.1)
Peripheral vascular disease, n (%)	10 (17.0)	28 (19.9)	8 (11.8)
Other cardiovascular diseases, n (%)	6 (10.2)	39 (27.7)	7 (10.3)
Charlson comorbidity sum score[d], median (Q1-Q3)	4 (3-4)	5 (3-6)	3 (2-4)
Duration of smoking >20 years, n (%)	43/50 (86.0)	107/120 (89.2)	56/59 (94.9)
Smoking >20 cigarettes per day, n (%)	6/48 (12.5)	20/120 (16.7)	12/59 (20.3)
Living with a smoking partner, n (%)	12/50 (24.0)	25/120 (20.8)	23/59 (39.0)
Motivation to quit[e], median (Q1-Q3)[b]	10 (8-10)	7 (5-9)	8 (6-10)
Readiness for smoking cessation[f]			
Preparation phase, n (%)	31/46 (67.4)	30/120 (25.0)	25/57 (43.9)
Pre-contemplation phase, n (%)	7/46 (15.2)	49/120 (40.8)	16/57 (28.1)
Nicotine dependency[g], median score (Q1-Q3)[d]	3 (2-5)	4 (2-6)	4 (3-5)
Type D personality[h], n (%)	5/30 (16.7)	16/89 (18.0)	9/48 (18.8)
Insomnia[i], n (%)	14/38 (36.8)	39/84 (46.4)	25/50 (50.0)
Average duration of sleep in hours, median (Q1-Q3)	7 (6-8)	7 (6-7.5)	7 (6-8)
HADS – anxiety and/or depression score ≥8, n (%)	11/39 (28.2)	39/101 (38.6)	16/50 (32)
Anxiety and/or affective disorder (hospital record diagnosis), n (%)	4 (6.8)	28 (19.9)	17 (25.0)
Travel distance in kilometers[j], median (Q1-Q3)	6.8 (3.7-13.1)	8.8 (3.8-14.5)	5.5 (2.7-12.1)
Inclusion in June or July[k], n (%)	6 (10.2)	18 (12.8)	13 (19.1)
Participation in CR, n (%)	14/56 (25.0)	13/98 (13.3)	17/60 (28.3)

Q1-Q3 = 25th-75th percentile, HADS: Hospital Anxiety and Depression Scale, CR: Cardiac Rehabilitation.

For variables with missing values, the fraction of respondents is presented.

[a]Low education was defined by completion of primary or secondary school only.

[b]Coronary heart disease is defined as angina or myocardial infarction.

[c]Cerebrovascular disease is defined as stroke or transient ischemic attack.

[d]Charlson comorbidity sum score, a scoring system to quantify a patient's somatic comorbidity and to predict a 10-year survival rate, with higher scores reflecting higher morbidity and mortality risk.

[e]Motivation to quit was assessed using a Likert scale ranging from 0 (low motivation) to 10 (high motivation).

[f]Readiness to quit was measured using a modified stage of change algorithm, categorizing individuals into precontemplation (no intent to quit within 6 months), contemplation (thinking about quitting in the next 6 months), or preparation (intends to quit in the next 30 days with a past quit attempt of at least 24 h).

[g]Nicotine dependency was assessed by Fagerströms test, a six-item questionnaire with scores ranging from 0 to 10: very low = 0-2, low = 3-4, moderate = 5, high = 6-7 and very high = 8-10.

[h]Type D personality was measured by the DS-14 questionnaire.

[i]Insomnia was measured by Bergen Insomnia Scale.

[j]Travel distance along the road network to the nearest municipal smoking cessation program serving the patients' area.

[k]Study inclusion in June or July, when summer closure or reduced staffing may delay initial contact from the smoking cessation program.

Supp. Table 3. Odds ratios for participation in the municipal smoking cessation program in the total population (excluding patients who quit smoking without program participation) from two randomized smoking cessation trials, six months after hospitalization for a vascular disease event, Norway, 2021-2023 (n = 209)

Variable	Crude		Adjusted for age, site and group allocation	
	OR (95% CI)	p-value	OR (95% CI)	p-value
**Patient factors**				
Age per 10 years	0.59 (0.43-0.81)	0.001	0.64 (0.44-0.92)[a]	0.016[a]
Female gender	1.57 (0.87-2.84)	0.132	1.25 (0.63-2.50)	0.529
Living alone	0.40 (0.20-0.80)	0.009	0.21 (0.09-0.49)	<0.001
Low education[b]	0.47 (0.23-0.96)	0.037	0.36 (0.15-0.86)	0.021
Employed	1.51 (0.77-2.96)	0.235	0.70 (0.29-1.69)	0.422
Charlson comorbidity score[c] per 1.0 point	0.71 (0.60-0.84)	<0.001	0.72 (0.56-0.93)	0.011
MI or stroke as index diagnosis	1.82 (1.01-3.28)	0.046	1.21 (0.60-2.42)	0.593
Motivation to quit smoking per 1.0 point[d]	1.19 (1.04-1.36)	0.014	1.13 (0.96-1.32)	0.140
Moderate to very high nicotine dependency[e]	0.76 (0.38-1.52)	0.435	0.77 (0.33-1.76)	0.533
Insomnia[f]	1.15 (0.57-2.33)	0.689	0.99 (0.43-2.32)	0.989
Shorter sleep duration (hours)	0.94 (0.73-1.20)	0.590	0.84 (0.62-1.14)	0.261
Type D personality[g]	1.05 (0.43-2.60)	0.911	1.00 (0.35-2.87)	0.998
HADS anxiety and/or depression score ≥8	0.75 (0.37-1.53)	0.427	0.65 (0.27-1.54)	0.323
Anxiety and/or affective disorder (hospital record diagnosis)	1.35 (0.68-2.68)	0.398	1.18 (0.51-2.73)	0.708
**Healthcare system factors**				
Travel distance[h] per 10 km	0.81 (0.59-1.11)	0.193	0.76 (0.53-1.10)	0.141
Inclusion in June/July[i]	1.62 (0.74-3.53)	0.229	1.99 (0.78-5.08)	0.151
Participation in CR	2.59 (1.15-5.81)	0.022	1.71 (0.63-4.66)	0.296

MI: Myocardial Infarction, HADS: Hospital Anxiety and Depression Scale, CR: Cardiac Rehabilitation.

Reference categories: female vs male, living alone vs with others, low vs high education, employed vs unemployed, MI/stroke vs other vascular index diagnoses, moderate to very high vs low nicotine dependency, insomnia (yes/no), type D personality (yes/no), HADS anxiety and/or depression score ≥8 vs <8, anxiety and/or affective disorder (yes/no), inclusion in June/July vs other months, participation in CR (yew/no).

[a]Adjusted for site and group allocation

[b]Low education was defined by completion of primary or secondary school only.

[c]Charlson comorbidity sum score, a scoring system to quantify a patient's somatic comorbidity and to predict a 10-year survival rate, with higher scores reflecting higher morbidity and mortality risk.

[d]Motivation to quit was assessed using a Likert scale ranging from 0 (low motivation) to 10 (high motivation).

[e]Nicotine dependency was assessed by Fagerströms test, a six-item questionnaire with scores ranging from 0 to 10: very low = 0-2, low = 3-4, moderate = 5, high = 6-7 and very high = 8-10.

[f]Insomnia was measured by Bergen Insomnia Scale

[g]Type D personality was measured by the DS-14 questionnaire.

[h]Travel distance along the road network to the nearest municipal smoking cessation program serving the patients' area.

[i]Inclusion in June or July, when summer closure or reduced staffing may delay initial contact from the smoking cessation program.

Supp. Table 4. Odds ratios for participation in the municipal smoking cessation program among the intervention group from two randomized smoking cessation trials, six months after hospitalization for a vascular disease event, Norway, 2021-2023 (n = 134)

Variable	Crude		Adjusted for age and site	
	OR (95% CI)	p-value	OR (95% CI)	p-value
**Patient factors**				
Age per 10 years	0.54 (0.36-0.81)	0.003	0.56 (0.37-0.85)[a]	0.006[a]
Female gender	1.27 (0.64-2.53)	0.498	1.25 (0.59-2.64)	0.555
Living alone	0.39 (0.18-0.85)	0.018	0.34 (0.14-0.82)	0.016
Low education[b]	0.75 (0.31-1.83)	0.527	0.80 (0.29-2.20)	0.660
Employed	1.18 (0.54-2.58)	0.687	0.45 (0.16-1.26)	0.127
Charlson comorbidity score[c] per 1.0 point	0.71 (0.57-0.89)	0.003	0.80 (0.61-1.06)	0.117
MI or stroke as index diagnosis	1.25 (0.63-2.49)	0.520	1.10 (0.52-2.36)	0.799
Motivation to quit smoking per 1.0 point[d]	1.11 (0.93-1.31)	0.258	1.05 (0.88-1.27)	0.587
Moderate to very high nicotine dependency[e]	0.91 (0.39-2.14)	0.826	0.58 (0.22-1.53)	0.270
Insomnia[f]	1.27 (0.54-3.02)	0.588	1.04 (0.41-2.64)	0.937
Shorter sleep duration (hours)	0.81 (0.59-1.10)	0.175	0.73 (0.51-1.03)	0.073
Type D personality[g]	0.85 (0.28-2.52)	0.765	0.79 (0.24-2.54)	0.687
HADS anxiety and/or depression score ≥8	0.88 (0.36-2.13)	0.770	0.62 (0.23-1.69)	0.352
Anxiety and/or affective disorder (hospital record diagnosis)	1.84 (0.76-4.48)	0.179	1.31 (0.49-3.47)	0.592
**Health care system factors**				
Travel distance[h] per 10 km	0.75 (0.53-1.08)	0.126	0.77 (0.52-1.13)	0.185
Inclusion in June/July[i]	1.32 (0.52-3.37)	0.556	1.34 (0.49-3.62)	0.567
Participation in CR	1.88 (0.74-4.75)	0.183	1.15 (0.41-3.21)	0.795

MI: Myocardial Infarction, HADS: Hospital Anxiety and Depression Scale, CR: Cardiac Rehabilitation.

Reference categories: female vs male, living alone vs with others, low vs high education, employed vs unemployed, MI/stroke vs other vascular index diagnoses, moderate to very high vs low nicotine dependency, insomnia (yes/no), type D personality (yes/no), HADS anxiety and/or depression score ≥8 vs <8, anxiety and/or affective disorder (yes/no), inclusion in June/July vs other months, participation in CR (yew/no).

[a]Adjusted for site.

[b]Low education was defined by completion of primary or secondary school only.

[c]Charlson comorbidity sum score, a scoring system to quantify a patient's somatic comorbidity and to predict a 10-year survival rate, with higher scores reflecting higher morbidity and mortality risk.

[d]Motivation to quit was assessed using a Likert scale ranging from 0 (low motivation) to 10 (high motivation).

[e]Nicotine dependency was assessed by Fagerströms test, a six-item questionnaire with scores ranging from 0 to 10: very low = 0-2, low = 3-4, moderate = 5, high = 6-7 and very high = 8-10.

[f]Insomnia was measured by Bergen Insomnia Scale.

[g]Type D personality was measured by the DS-14 questionnaire.

[h]Travel distance along the road network to the nearest municipal smoking cessation program serving the patients' area.

[i]Study inclusion in June or July, when summer closure or reduced staffing may delay initial contact from the smoking cessation program.

Supp Table 5. Cross-table of correlations between patient- and healthcare system factors in the total population from two randomized smoking cessation trials among patients hospitalized for a vascular disease event, Norway, 2021-2023 (n = 268)

	Age per 10 years	Female gender	Low education	Employed	Living alone	MI/stroke as index diagnosis	Charlson comorbidity	Smoking partner	Baseline motivation	Prepared for cessation	Pre-contemplation	Nicotine dependency	HADS anxiety ≥8	HADS depression ≥8	Type D personality	Insomnia	Shorter sleep duration	Anxiety disorder	Affective disorder	Travel distance to HLC	Study inclusion in June/July	Participation in CR
Age per 10 years	1																					
Female gender	0.03	1																				
Low education	0.03	0.04	1																			
Employed	-0.48**	0.00	-0.08	1																		
Living alone	-0.04	0.18**	0.08	-0.01	1																	
MI/stroke as index diagnosis	-0.07	-0.02	-0.03	0.15*	-0.14*	1																
Charlson comorbidity score per 1.0 point	0.66**	-0.02	0.11	-0.41**	0.02	-0.05	1															
Living with a smoking partner	-0.10	-0.17*	0.04	-0.03	-0.38**	-0.00	-0.05	1														
Baseline motivation per 1.0 point	-0.17*	-0.04	-0.06	0.24**	-0.10	0.18**	-0.23**	0.02	1													
Prepared for cessation	-0.06	-0.03	-0.07	0.11	-0.10	0.14*	-0.12	-0.02	0.50**	1												
Precontemplation	0.21**	0.08	0.12	-0.22**	0.11	-0.09	0.22**	-0.00	-0.63**	-0.55**	1											
Moderate to very high nicotine dependency	-0.19**	0.01	-0.08	0.13	-0.04	0.04	-0.05	0.07	-0.03	-0.07	-0.01	1										
HADS anxiety ≥8	-0.10	0.00	-0.01	-0.06	0.02	0.02	0.02	0.13	-0.16*	-0.07	0.06	0.14	1									
HADS depression ≥8	-0.06	0.12	-0.01	-0.11	0.16*	0.03	0.12	-0.03	-0.11	-0.06	0.09	0.11	0.56**	1								
Type D personality	-0.18*	-0.06	-0.06	0.07	0.01	0.03	0.01	0.04	0.03	0.05	-0.10	0.07	0.45**	0.54**	1							
Insomnia	-0.10	0.02	-0.15	0.09	0.01	0.06	0.00	0.07	0.00	-0.04	-0.04	0.19*	0.29**	0.25**	0.23**	1						
Shorter sleep duration (hours)	-0.21**	-0.00	0.06	0.13	-0.07	0.00	-0.03	0.10	-0.11	-0.10	0.05	0.01	0.15	0.06	-0.00	0.27**	1					
Anxiety disorder	-0.23**	0.06	-0.01	-0.03	0.13	-0.01	-0.14*	0.04	-0.06	-0.10	0.07	0.21*	0.29**	0.15*	0.15*	0.12	-0.01	1				
Affective disorder	-0.20**	0.07	0.04	-0.07	0.08	0.01	-0.09	-0.01	-0.04	-0.10	0.14*	0.09	0.18*	0.16*	0.15	0.12	-0.10	0.65**	1			
Travel distance to HLC in kilometers	-0.00	-0.13*	0.10	-0.03	-0.06	-0.03	0.01	0.07	0.01	-0.06	0.00	-0.03	-0.06	-0.10	-0.05	-0.12	0.10	-0.08	-0.12	1		
Study inclusion in June/July	0.05	0.01	-0.02	-0.18**	0.05	-0.04	0.04	-0.08	-0.01	0.08	-0.07	-0.10	-0.07	-0.01	0.01	0.01	-0.17*	-0.05	0.00	0.01	1	
Participation in CR	-0.26**	-0.01	-0.07	-0.00	-0.02	0.23**	-0.19**	0.14	0.14	0.12	-0.11	-0.02	0.01	0.11	0.16	0.04	0.06	-0.03	-0.01	0.01	0.07	1

* = < 0.05, ** = < 0.01.

Supp table 6. Odds ratios for participation in the municipal smoking cessation program in the total population (excluding total non-response to the questionnaire) from two randomized smoking cessation trials, six months after hospitalization for a vascular disease event, calculated with the use of multiple imputation, Norway, 2021-2023 (n = 233)

**Variable**	**Crude**		**Adjusted for age, site and group allocation**	
	OR (95% CI)	p-value	OR (95 % CI)	p-value
Living alone	0.50 (0.26-0.97)	0.041	0.31 (0.14-0.67)	0.003
Low education[a]	0.48 (0.25-0.95)	0.034	0.41 (0.18-0.92)	0.030
Employed	1.20 (0.64-2.26)	0.565	0.53 (0.23-1.22)	0.136
Motivation to quit smoking per 1.0 point[b]	1.07 (0.95-1.21)	0.263	1.00 (0.87-1.16)	0.960
Moderate to very high nicotine dependency[c]	1.09 (0.58-2.02)	0.795	1.00 (0.48-2.07)	0.988
Insomnia[d]	1.29 (0.68-2.42)	0.436	1.17 (0.56-2.42)	0.679
Shorter sleep duration (hours)	0.99 (0.79-1.24)	0.911	0.95 (0.73-1.24)	0.721
Type D personality[e]	1.14 (0.50-2.57)	0.756	0.96 (0.38-2.41)	0.935
HADS anxiety and/or depression score ≥8	1.07 (0.56-2.03)	0.850	0.91 (0.43-1.94)	0.815
Participation in CR	1.59 (0.78-3.22)	0.199	1.21 (0.52-2.80)	0.657

HADS: Hospital Anxiety and Depression Scale, CR: Cardiac Rehabilitation.

Reference categories: living alone vs with others, low vs high education, employed vs unemployed, moderate to very high vs low nicotine dependency, insomnia (yes/no), type D personality (yes/no), HADS anxiety and/or depression score ≥8 vs <8, participation in CR (yew/no).

[a]Low education was defined by completion of primary or secondary school only.

[b]Readiness to quit was measured using a modified stage of change algorithm, categorizing individuals into precontemplation (no intent to quit within 6 months), contemplation (thinking about quitting in the next 6 months), or preparation (intends to quit in the next 30 days with a past quit attempt of at least 24 h).

[c]Nicotine dependency was assessed by Fagerströms test, a six-item questionnaire with scores ranging from 0 to 10: very low = 0-2, low = 3-4, moderate = 5, high = 6-7 and very high = 8-10.

[d]Insomnia was measured by Bergen Insomnia Scale.

[e]Type D personality was measured by the DS-14 questionnaire.
